# Variability analysis of muscle activation symmetry to identify indicators of individual motor strategy: a case series on elite Paralympic powerlifters

**DOI:** 10.3389/fspor.2023.1290964

**Published:** 2023-11-03

**Authors:** Lorenzo Rum, Ruggero Romagnoli, Aldo Lazich, Tommaso Sciarra, Nicoletta Balletti, Maria Francesca Piacentini, Alessandro Boraschi, Elena Bergamini

**Affiliations:** ^1^ Department of Movement, Human and Health Sciences, University of Rome “Foro Italico”, Rome, Italy; ^2^ Department of Biomedical Sciences, University of Sassari, Sassari, Italy; ^3^ Defense Veterans Center, Celio Army Medical Center, Rome, Italy; ^4^ Department of Computer, Control and Management Engineering, University of Rome “Sapienza”, Rome, Italy; ^5^ STAKE Lab, University of Molise, Pesche, Italy; ^6^ Para Powerlifting Section, Italian Weightlifting Federation ‘FIPE’, Rome, Italy

**Keywords:** disability sport, Paralympic powerlifting, bench press, wearable technology, asymmetry, individual muscle strategy

## Abstract

**Introduction:**

In Paralympic powerlifting competitions, movement execution symmetry is a technical requirement influenced by individual athlete characteristics and motor strategies. Identifying the elements associated with individual motor strategies can offer valuable insight for improving sport performance. Therefore, this case series study aimed to explore muscle activation symmetry and its intra- and inter-individual variability to determine the muscles mostly related to individual motor strategies in elite Paralympic powerlifters.

**Methods:**

Bilateral electromyographic activation of the anterior deltoid (AD), pectoralis major (PM), latissimus dorsi (LD), triceps (TRI) and external oblique (EO) muscles were analysed in five elite Paralympic powerlifters while performing four sets of one-repetition maximum of Paralympic bench press. Muscle activation symmetry indexes (SI) were obtained and transformed to consider individual-independent evaluation. The coefficient of variation (CV), variance ratio (VR), and mean deviation (MD) were computed to assess inter- and intra-individual variability in electromyographic waveforms and SI.

**Results:**

Both transformed and non-transformed SI indicated overall symmetric activation in DA, PM, TRI, and LD. Transformed SI revealed asymmetrical muscle activation of EO when grouping data (mean bilateral difference: 10%). Athletes exhibited low intra-individual SI variability in all analysed muscles (CV < 10%) and low inter-individual variability in DA, PM, LD, and TRI (CV < 10%; VR: 4%–11%; MD: 29%–43%). In contrast, higher inter-individual variability was observed in EO (CV: 23%; VR: 23%; MD: 72%–81%).

**Conclusion:**

The highest variability and asymmetry in abdominal muscle activation among athletes emphasize the importance of personalized training approaches for targeting these muscles due to their role in individualizing motor strategies.

## 1. Introduction

The increasing participation of individuals with disabilities in sports, driven by societies' increased awareness of their capabilities and potential, represents a significant step towards inclusivity and empowerment. In this context, Paralympic powerlifting has witnessed a remarkable surge in participation and competitive accomplishments at the international level, becoming one of the fastest-growing sports within the Paralympic movement (1, 2). This positive trend aligns with the increasing scientific research activity in recent years, which has delved into the biomechanical, physiological, and psychological aspects of this Paralympic discipline (3). Despite this, certain aspects that are more tuned to individual differences among para-athletes, such as impairment type, remain underrepresented in research (4–7). Research in this direction is crucial as it provides valuable insights for optimizing personalised training programs and promoting the long-term success and well-being of individual athletes.

Paralympic powerlifting revolves around a single exercise, namely, a maximal bench press lift (one repetition maximum, 1RM) on a flat bench. In competition, a critical technical requirement is the symmetrical execution of the lift, and not meeting this criterion can impact the validity of the attempt (8). Owing to its significance in competition and sport performance, the assessment of bilateral symmetry in the lift has been previously examined from multiple perspectives, including barbell and arm kinematics, as well as muscle activity, in athletes with and without disability (9–13). The findings of these studies indicate that Paralympic powerlifters exhibit overall movement symmetry, but asymmetries tend to be more pronounced at higher loads (∼90% 1RM). Furthermore, recent research has suggested that fatigue, even at lower sub-maximal exercise intensity (80% 1RM), can differentially affect the muscle activity between dominant and non-dominant sides in shoulder muscles (e.g., pectoralis muscle), accentuating the likelihood of asymmetry (13).

The presence of asymmetrical muscle strength and activation is frequently associated with increased injury risk (14) and reduced athletic performance (15), although research outcomes are not always consistent (16, 17). Muscle strength and activation asymmetry can stem from various factors, such as the specific muscle investigated (18), limb dominance (19), and training habits (11). In the specific context of Paralympic athletes, additional contributors to asymmetry could include the impact of disability on anatomical side differences, such as leg length differences in amputees, and neuromuscular imbalances resulting from neurological conditions (20). Research studies on muscle activation symmetry in Paralympic bench press reported conflicting results, with both asymmetrical (9) and symmetrical (10, 13) activation of shoulder and arm muscles being observed. These contrasting outcomes may be linked not only to the small sample size and the different computational approaches to symmetry evaluation, but also to the heterogeneity of impairment types among the included athletes. Supporting this hypothesis, a previous study comparing Paralympic powerlifters with and without spinal cord injury found distinct muscular activation patterns between the two groups, suggesting a potential impact of individual disability/impairment type (6). However, the symmetry of muscle activation pattern was not investigated, nor was its variability.

The analysis of variability in muscle activation can provide crucial information about the central organization of motor strategies and motor skill acquisition (21). For instance, previous study on elite bench press performance indicated a more individualized motor strategy among experienced powerlifters evidenced by an increased inter-individual variability in muscle coordination (22). However, it's worth noting that the existing literature lacks a comprehensive understanding of the potential indicators of individual motor strategy, particularly in terms of identifying which muscle can provide the most informative insights. Distinct individual motor strategies and their potential association with specific muscle groups can be identified by leveraging intra- and inter-individual variability analysis, yielding useful knowledge for optimizing personalised training programs and understanding/preventing potentially risky situations.

Therefore, in this case series study, we analysed the symmetry of muscle activation and its inter- and intra-individual variability with the aim of identifying which muscle group is mostly related to individual motor strategies (i.e., with low intra-individual variability but high inter-individual variability) during maximal Paralympic bench press in elite athletes. We hypothesised that athletes would demonstrate individual motor strategies characterised by low intra-individual variability of muscle activation symmetry, whereas we expect to observe high inter-individual variability in the muscles that play a crucial role in compensating for individual impairments.

## 2. Materials and methods

### 2.1. Study design and setting

This case series study involved testing procedures performed in a single experimental session at the Italian Army Sports Center (Rome, Italy) during the first day of a mid-season weekend retreat (from 10:00 a.m. to 20:00 p.m.). Considering the context of testing within a scheduled seasonal retreat, athletes were instructed to optimize their physical condition by tailoring their personal habits and training programs to align with the retreat. Additionally, consistent environmental conditions of the setting on the testing day were ensured through air conditioning. The experimental protocol was approved by the institutional review board of the University of Rome “Foro Italico” (CAR 116/2022), and all participants provided written informed consent before testing.

### 2.2. Participants

The study involved five international-level elite Paralympic powerlifters (age: 30.0 ± 5.1 years, body mass: 74.1 ± 17.1 kg, males) (Table 1). To be included in the study, participants needed to be eligible for Paralympic powerlifting competition (20) and to achieve the minimum qualification standards for World Championships and Paralympic Games (23). The exclusion criterium for the study was the presence of impairment or injury in the upper limbs on the day of testing.

**Table 1:** Individual athlete data.

Athlete	Age (years)	Sitting height (cm)	Body mass (kg)	Disability	1RM (kg)
#1	32	78	101	Unilateral transtibial amputation (left)	192
#2	37	76	71.8	Unilateral transfemoral amputation (left)	155
#3	30	64	54.5	Spina bifida	133
#4	23	77	76	Bilateral transfemoral amputation	200
#5	28	64	67	Spina bifida	132

RM, repetition maximum.

### 2.3. Data measurement

Participants performed a standardized warmup based on the most recent competition result of 1RM (6 repetitions at 40% 1RM, 3 repetitions at 60% 1RM, 2 repetitions at 75% 1RM, and 1 repetition at 85% 1RM) with three-minute rest pauses between sets (24). After the warmup, data from four attempts of 1RM Paralympic bench press were collected with five-minute rest pauses between attempts to mitigate the impact of fatigue. The athlete's recovery was monitored using the Total Quality Recovery scale before proceeding with the next attempt (25). The first attempt was based on the athlete's most recent 1RM competition result. Experienced technical personnel were present for assistance in case of failure to minimize the risk of injury. Body positioning on the Paralympic powerlifting bench (Eleiko, Halmstad, Sweden) was standardized during each lift and adhered to official technical regulations, involving the use of leg straps to secure the lower body to the bench, while no exceptions were made for lower and upper body positioning or elbow angle (8). The data analysis considered only lifts that were performed with the correct lift sequence, in accordance with official regulations (8). This determination was made on-site and verified through video recordings by three coaches possessing international experience. After complete recovery from 1RM trials (at least 5 min), the athlete performed two maximal voluntary isometric contraction (MVIC) tests that were used for normalising the EMG data (26). During these trials, the athlete was positioned on the bench exerting a maximal pushing force against the barbell (Eleiko, Halmstad, Sweden) which was appropriately locked to obtain a 90° angle at the elbow and a sternum-to-bar distance of approximately 15 cm (6). Each MVIC test lasted 5 s with a 5-min break between trials (please refer to Supplementary Figure S1 for the entire experimental protocol).

The barbell trajectory on the frontal plane was estimated through a high-resolution and high-speed camera (Hero 9, GoPro, San Mateo, CA, USA, sampling frequency: 120 Hz). The action camera was positioned on a tripod at 1-m height from the floor and 1-m away from the rear of the bench, perpendicularly to the barbell longitudinal axis, to record the 2D position of two reflective markers. The two markers (diameter 17 mm) were placed laterally on the barbell endcaps. The marker's vertical displacement was obtained using the open-source software Kinovea (version 0.9.5) (12, 27). To minimize depth errors in the sampled data, the known length of the bar in the picture frame immediately before the exercise execution was used for software calibration. A 600% zoom was used for the frame-by-frame visual inspection to enhance the precision of the marker tracking and manual corrections were made whenever necessary.

The arm and upper body muscle activity was bilaterally measured by surface EMG (MiniWave, Cometa, Bareggio, Italy, sampling frequency: 2,000 Hz). Surface electrodes (Ag/AgCl, 20 mm inter-electrode distance) were placed on the anterior deltoid (AD), sternal portion of pectoralis major (PM), latissimus dorsi (LD), long head of the triceps (TRI) and external oblique of the abdomen (EO). To improve recorded signal quality, SENIAM recommendations were taken as a reference for skin preparation as well as electrode placement and fixation procedures (28). The video recordings and EMG sensor systems were synchronised using the EMG acquisition software (EMGandMotionTools, version 8.6.2.0, Cometa, Bareggio, Italy).

### 2.4. Variables

The symmetry of movement execution was analysed on kinematic and muscle activation variables. The barbell vertical velocity was derived from the markers vertical displacement after smoothing the video recorded signal with a 20 Hz second-order Butterworth filter (12). A previously validated algorithm was used to identify the timings of the main events of the Paralympic bench press lift (i.e., initial bar lowering, and lift end at complete elbow extension) and to segment barbell kinematic and muscle activation data (29, 30). Briefly, this algorithm exploits the features of the barbell vertical velocity profile and detects significant changes in the curve slope which are known to be associated with specific events of the lift (e.g., initial bar lowering: initial decline of velocity from zero to negative values). The lift cycle was then normalised to 100 data points including both eccentric and concentric phases.

EMG raw data was band-pass filtered with a 10–500 Hz second-order Butterworth filter, full-wave rectified, and smoothed with a 10 Hz fourth-order Butterworth filter to obtain a linear envelope. For each muscle, the maximum value between EMG root mean squares computed over 3 s of maximal muscle activation in the two MVIC trials was used for normalisation. The amplitude-normalised signal was then integrated over the entire lift cycle to compute the following symmetry index (SI):

$$\mathrm{SI}=\;\frac{\int_{i=1}^{k}\mathrm{EM}G_{\mathrm{right}}}{\left(\int_{i=1}^{k}\mathrm{EM}G_{\mathrm{right}}+\;\int_{i=1}^{k}\mathrm{EM}G_{\mathrm{left}}\right)}*100$$

where *k* is the number of time intervals of the lift cycle. SI of 50% indicates perfect symmetry in muscle activation between sides and values >50% represent a greater contribution of right side (31). In addition, SI was transformed by subtracting 50% and then taking the absolute value. The transformed SI ranged 0%–50%, with 0 value indicating perfect symmetry in bilateral muscle activation. This methodological approach was adopted to consider differences in dominant and/or impaired side between individuals and facilitate the comparison of results among athletes.

Both intra- and inter-individual variability of muscle activation symmetry were assessed using the coefficient of variation (CV) of non-transformed SI (32). In addition, for quantifying inter-individual variability, the variance ratio (VR) and mean deviation (MD) were also calculated from right and left EMG curves over the complete lift cycle as in (33). These two metrics are widely used in the assessment of inter-individual variability and were added to overcome the limitation of the CV (32, 33). The CV is influenced by the mean EMG value (at the denominator in the CV formula), potentially overestimating variability when muscle activity is weak or inactive (33). In this regard, the VR and MD are less influenced by mean EMG values and provide a good measure of repeatability in waveform shapes (33, 34).

## 3. Results

**Figure 1:**
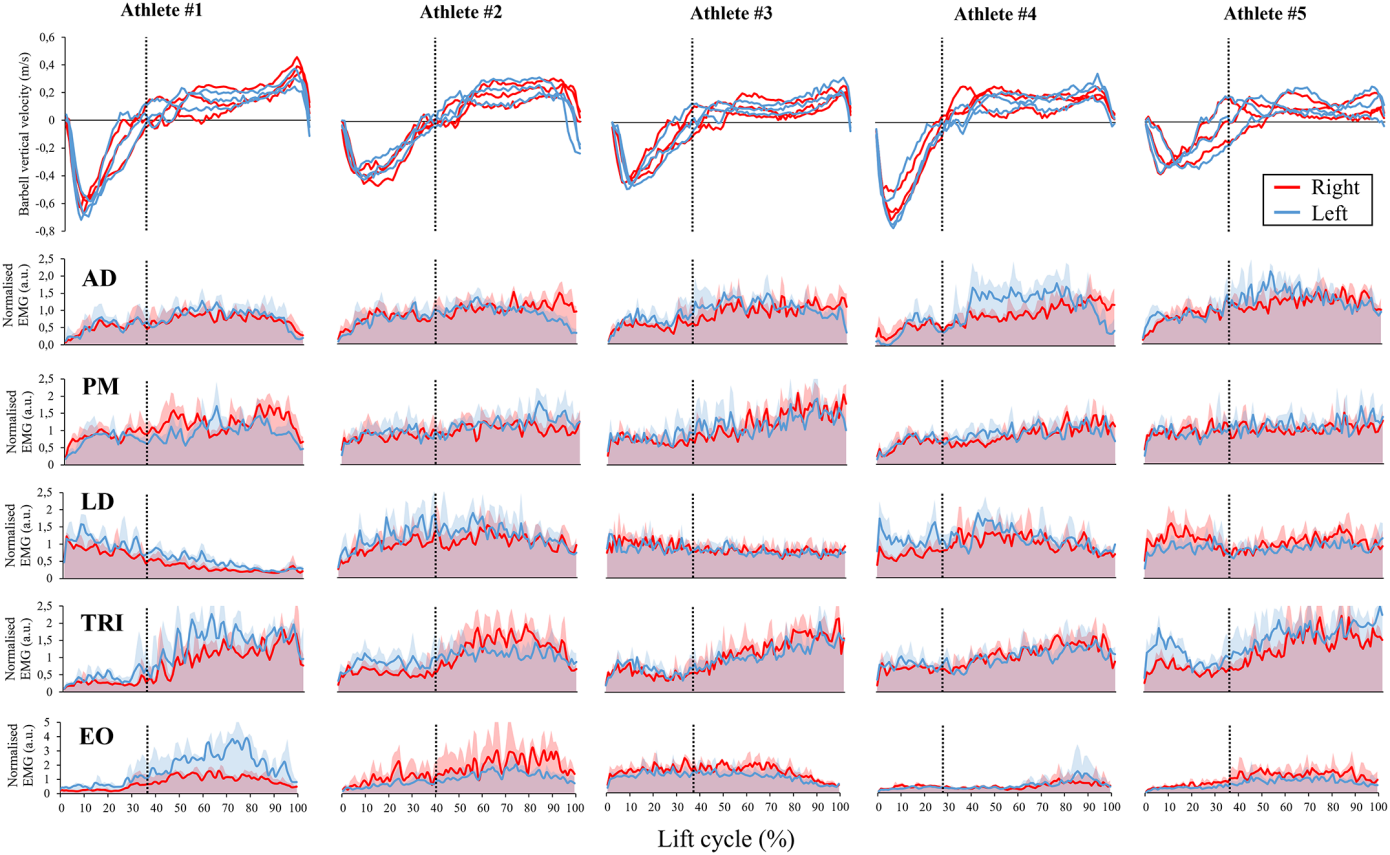
Right (red) and left (blue) barbell vertical velocity profiles (top panels) and muscle activity envelopes normalised over the lift cycle for each athlete. In the top row of the panel, the barbell velocity profiles of single valid lifts are reported. In the following rows, the EMG envelopes of anterior deltoid (AD), pectoralis major (PM), latissimus dorsi (LD), triceps (TRI), and external oblique (EO) are reported as individual mean (solid line) and standard deviation (shaded area). Vertical dashed lines represent the individual average timing of barbell stop at chest, marking the transition between the eccentric and concentric phases. The area under the EMG curves was shaded to emphasize both overlapping muscle activation between the two sides and asymmetric activations.

Out of the four 1RM attempts made by the athletes, four athletes achieved three valid lifts, while one athlete successfully completed all four lifts. For this athlete, only results from the last three valid trials are presented. Figure 1 shows the bilateral barbell velocity profiles and EMG curves over the lift cycle per each athlete. Despite individual small variations, all athletes exhibited progressively increasing activity of AD, PM, and especially TRI, along the lift execution. Higher muscle activation bursts were observed during the concentric phase (later positive barbell vertical velocity in the top panel of Figure 1) compared to the eccentric phase (initial negative velocity).

### 3.1. Symmetry of muscle activity

Table 2 shows the non-transformed and transformed SI for each athlete. In general, individual values indicated good symmetry (non-transformed SI: 45%–55%, transformed SI: 0%–5%) in AD, PM, LD, and TRI, with only two exceptions. Athlete 1 displayed a higher contribution from left-side LD and TRI (non-transformed SI < 45%), which corresponds to the same side as the unilateral amputation. Athlete 5 showed a similar higher muscle activity at left side in TRI.

**Table 2:** Symmetry indexes (mean ± SD) of bilateral muscle activation per each muscle (range 0–100%). Transformed symmetry indexes (range: 0%–50%) are also provided.

	Athlete #1	Athlete #2	Athlete #3	Athlete #4	Athlete #5	Group
SI (%)						
DA	48 ± 1	53 ± 1	47 ± 3	45 ± 5	48 ± 1	49 ± 4
PM	55 ± 2	47 ± 1	50 ± 1	49 ± 3	49 ± 1	50 ± 4
LD	42 ± 2*	45 ± 1	51 ± 2	45 ± 3	54 ± 1	47 ± 5
TRI	42 ± 1*	53 ± 5	51 ± 1	51 ± 2	43 ± 3*	48 ± 5
EO	31 ± 3*	63 ± 2*	58 ± 5*	49 ± 3	61 ± 2*	49 ± 12
Transformed SI (%)						
DA	2 ± 1	3 ± 1	3 ± 3	5 ± 4	2 ± 1	3 ± 3
PM	5 ± 2	3 ± 1	1 ± 0	3 ± 1	1 ± 1	3 ± 2
LD	8 ± 2*	5 ± 1	1 ± 1	5 ± 3	4 ± 1	4 ± 4
TRI	8 ± 1*	5 ± 3	1 ± 1	1 ± 2	7 ± 3*	4 ± 3
EO	19 ± 3*	13 ± 2*	8 ± 5*	2 ± 2	11 ± 2*	11 ± 6*

SI, symmetry index.

* >5% difference between sides.

When considering mean group values, non-transformed SI of each muscle were centred close to perfect symmetry (SI = 50%), while transformed SI revealed the presence of muscle activation asymmetry. The highest asymmetry was observed in EO as four out of five athletes reported transformed SI greater than 5% and up to 19% (maximal range 0%–50%). When observing the non-transformed SI values to assess the side of asymmetry, it was evident that athlete 1 exhibited a more significant contribution from the left EO muscle. On the other hand, athletes 2, 3, and 5 demonstrated higher muscle activity on their right side.

### 3.2. Intra- and inter-individual variability

**Figure 2:**
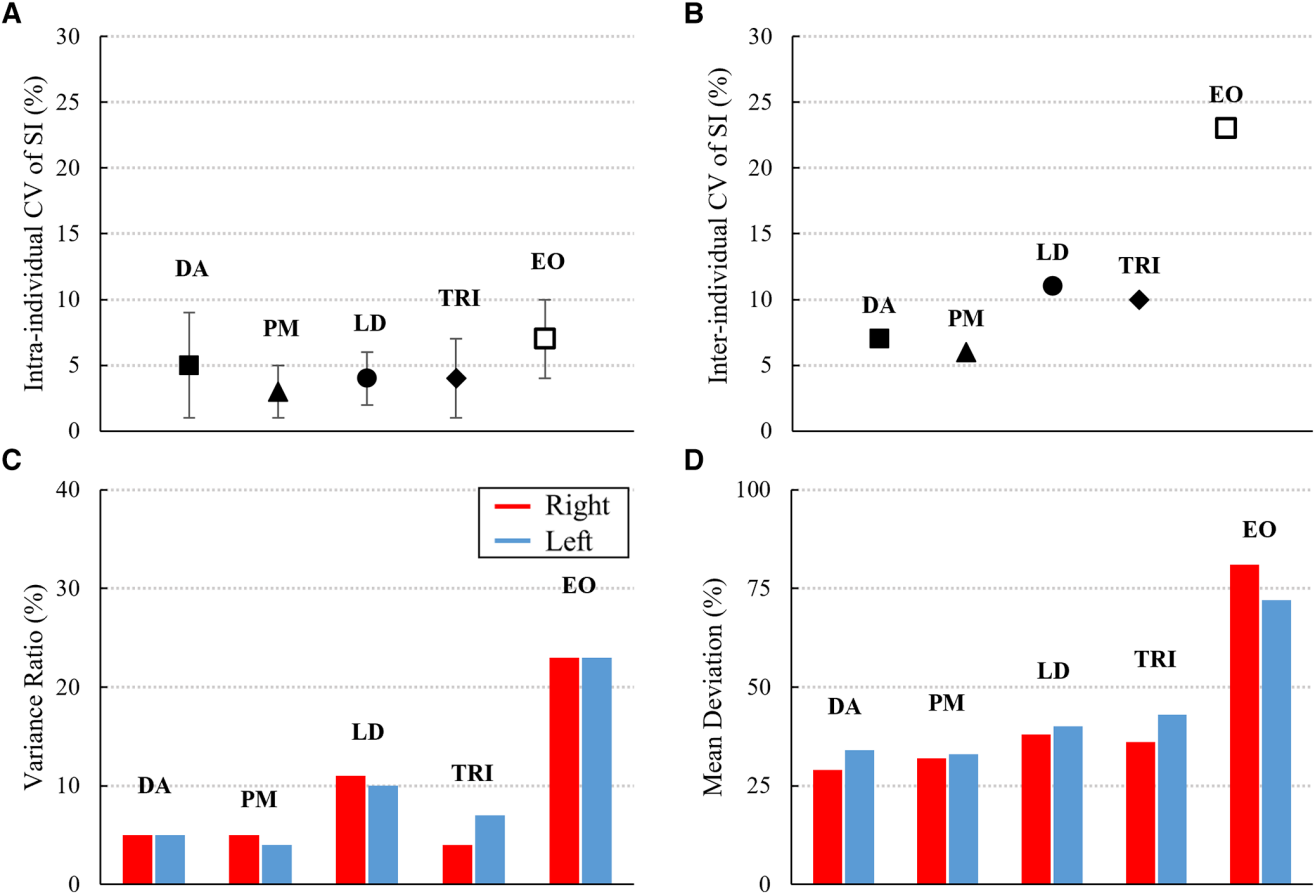
Variables of intra- and inter-individual variability of EMG signal: (**A,B**) intra- and inter-individual coefficient of variation (CV) of non-transformed symmetry index (SI, mean ± standard deviation); (**C,D**) variance ratio and mean deviation of right (red) and left (blue) EMG envelopes of anterior deltoid (AD), pectoralis major (PM), latissimus dorsi (LD), triceps (TRI), and external oblique (EO) during the complete lift cycle.

Athletes exhibited consistently low intra-individual variability in muscle activation symmetry, with the CV of non-transformed SI below 10% in each observed muscle (Figure 2A). Similar results were displayed for the inter-individual analysis of DA, PM, LD, and TRI muscles, where the CV of non-transformed SI was less than (or equal to) 10%, demonstrating low variability (Figure 2B). Conversely, the EO muscle displayed the highest variability among participants, with a CV of 23% (Figure 2B). Additionally, other measures of inter-individual variability of EMG curves over the complete lift cycle (VR and MD) revealed comparable findings, with DA, PM, LD, and TRI muscles demonstrating relatively consistent variability and the EO muscle showing the greatest variability among athletes (Figures 2C,D).

## 4. Discussion

The aim of this case series study was to identify the individual motor strategies and the muscle groups primarily linked to individual differences in elite Paralympic powerlifters through the analysis of muscle activation symmetry and its variability. The findings revealed that athletes displayed a consistent low intra-individual variability in muscle activation symmetry of all the observed muscles. Similarly, a low inter-individual variability was reported in the prime mover muscles involved in the lift, including deltoid anterior, pectoralis major, and triceps muscles. On the other hand, the muscles responsible for movement stabilisation, particularly the abdominal external oblique, demonstrated the highest variability among the participants.

The visual inspection of muscle activation patterns indicated that the motor strategy for the deltoid anterior, pectoralis major, and triceps muscles was similar across the participants. The anterior deltoid and pectoralis major were symmetrically (SI: 45%–55%) activated throughout the entire lift cycle, with a greater involvement during the concentric phase. A similar but more pronounced behaviour was displayed by the triceps muscle, whose peak of activation was reached at a later stage of the concentric phase and related to elbow extension. These findings align with previous research in both athletes with and without disability performing maximal bench press, confirming an increasing muscle activation in the deltoid anterior and pectoralis major muscles over the lift cycle, as well as a prominent activation of triceps around the sticking region of the concentric phase (35–37). Noteworthy, as indicated by the symmetry indexes, two athletes exhibited an asymmetrical activation of the triceps in the eccentric (athlete #5) and concentric (athlete #1) phase, respectively. By examining video recordings, we hypothesized that this phenomenon could be associated with rotational forces generated by asymmetric force transfer resulting from variations in lower body positioning and contact with the bench. Specifically, athlete #5 adopted an asymmetric leg placement due to motor impairment constraints, and difference in pressure against the bench between residual and prosthetic heels likely occurred in athlete #1. These rotational forces appeared then to transmit through the upper body, involving segments like the pelvis and upper trunk, ultimately affecting the arms. Unfortunately, the methodology adopted in this study lacked the necessary data, such as force/pressure measurements between the athletes and the bench, to comprehensively explain this phenomenon. Nevertheless, this perspective introduces a novel consideration regarding lower body positioning and physical impairment characteristics in the individualization of the muscle activation strategy in Paralympic bench press.

The symmetrical activation of shoulder and arm muscles, as indicated by the symmetry indexes, underscores their crucial role in the symmetrical execution of the bench press movement, a skill refined by elite athletes to meet competition requirements (8). Nevertheless, this observation also suggests that a singular focus on these muscles may not provide substantial insights for personalized evaluation approaches. On the contrary, the symmetry indexes revealed a higher degree of asymmetry in abdominal stabilizing muscles. This indicates that, while symmetry may be expected at the higher body levels performing the motor action and in the assessment of barbell kinematics, the stabilizing action of abdominal muscles may not necessarily exhibit the same level of symmetry. Nevertheless, when compared to previous literature, these results confirmed the reported absence of bilateral differences in shoulder and arm muscles (10, 13), although being in contrast with the asymmetries in the activation of pectoralis major and deltoid anterior muscles reported by Aedo-Munoz et al. (9). In this regard, it is worth to acknowledge that Aedo-Munoz and colleagues (9) compared EMG signals between sides without a normalization procedure, primarily due to experimental limitations. This approach is recognized for disregarding physiological and anatomical factors that might influence the amplitude of the EMG signal and its association with muscle activation (26). Irrespective of the different outcomes, instances of asymmetrical activation in shoulder and arm muscles were identified in both our investigation and previous study (9). This underscores the need for methodologies able to discern individual attributes in muscle activation symmetry.

Most of the previous studies relied on right/left or dominant/non-dominant comparison to assess muscle activation symmetry during the bench press lift (9, 10, 19, 38). However, this approach may not be the most appropriate when dealing with athletes with different disabilities, where the right/dominant or left/non-dominant upper body side may not correspond to the side affected by the impairment. To address this limitation, we employed a transformed symmetry index to obtain information about symmetry independent on the body side. Notably, when aggregating values across athletes, asymmetries were emphasized solely within the transformed symmetry index. This methodology allowed for a more effective variability analysis, aligning with the International Paralympic Committee's principle of developing impairment-independent tools for Paralympic performance evaluation (39, 40). As a result, we were able to identify low inter-individual variability of muscle activation symmetry in most investigated muscles and to highlight those muscles requiring further attention. As wearable EMG technologies continue to advance and become more feasible for in-field use (41–43), future studies are suggested to implement this methodological approach and evaluate its effectiveness in accounting for the potential impact of individual impairments on sport performance.

The analysis of variability measures revealed a consistent low intra-individual variability of muscle activation symmetry among all athletes and muscles. This outcome is likely related to the elite level of the participants, as higher levels of training experience are associated with more individualised and less variable motor strategies (22). When assessing inter-individual variability of both muscle activation symmetry (CV of non-transformed SI) and bilateral EMG curves (VR and MD), the shoulder and arm muscles were the less variable between athletes. This underscores the high degree of specialization among elite athletes who have refined a consistent symmetrical muscle activation pattern for the muscles primarily engaged in the bench press action (13). On the other hand, the abdominal external oblique muscle displayed a high inter-individual variability, indicating its potential association with individual motor strategy. Previous EMG studies on Paralympic bench press have primarily focused on the examination of shoulder and arm muscles, while the stabilising role of abdominal muscles has been inadequately explored (7, 35, 44, 45). This study accentuates the significance of understanding the activation strategies of these stabilizing muscles, especially given that prominent impairment types in Paralympic powerlifting pertain to lower limbs and necessitate robust trunk and abdominal motor control for stability. Furthermore, this is particularly significant as Paralympic athletes perform bench press while lying with their entire body on a flat bench without placing their legs to the ground for force transfer (8, 11). Therefore, addressing this gap in research is paramount to not only for improving performance and ensuring the safety of Paralympic powerlifters, but also to provide further insights on the relationship between impairment type and sport performance.

While this study offers insights into specific aspects of muscle activation symmetry among elite Paralympic powerlifters, the study's case series design inherently imposes limitations on the generalizability of the findings to a broader population. The constrained sample size, coupled with the diverse impairments and individual characteristics among the recruited athletes, may have amplified the heterogeneity within the sample. Consequently, this increased heterogeneity makes it challenging to conclusively extrapolate the results to a more diverse and extensive athlete population. Nonetheless, we observed a consistent low level of inter-individual variability in muscle activation symmetry across most investigated muscles, providing initial indicators and methodological suggestions. With a larger sample size, a more comprehensive statistical analysis could be conducted, potentially revealing nuanced patterns of muscle activation and asymmetry that are specific to various impairment types.

In conclusion, the main results of this study indicate that symmetrical and less variable muscle activity can be expected in arm and shoulder muscles but not in abdominal muscles in Paralympic powerlifting. Although being limited to a small sample size due to the study design, these findings emphasize the importance of addressing asymmetries to optimize training strategies and enhance overall performance in this sport. Abdominal muscles have been indicated as pivotal components in individual motor strategies for elite Paralympic powerlifters, revealing substantial variability and asymmetry. Understanding and targeting these muscle groups through tailored training protocols can be crucial for optimizing performance through the development of personalized training regimens for athletes in this discipline. By further exploring the underlying mechanisms of asymmetry and inter-individual variability, researchers and coaches can develop targeted interventions tailored to individual motor strategy for the support of the Paralympic athlete.

## Data Availability

The raw data supporting the conclusions of this article will be made available by the authors, without undue reservation.
