# Supplementary figures and images for: Variability analysis of muscle activation symmetry to identify indicators of individual motor strategy: a case series on elite Paralympic powerlifters
Source: Front Sports Act Living. 2023 Nov 3;5:1290964. doi: 10.3389/fspor.2023.1290964 (PMC10655026; doi:10.3389/fspor.2023.1290964)

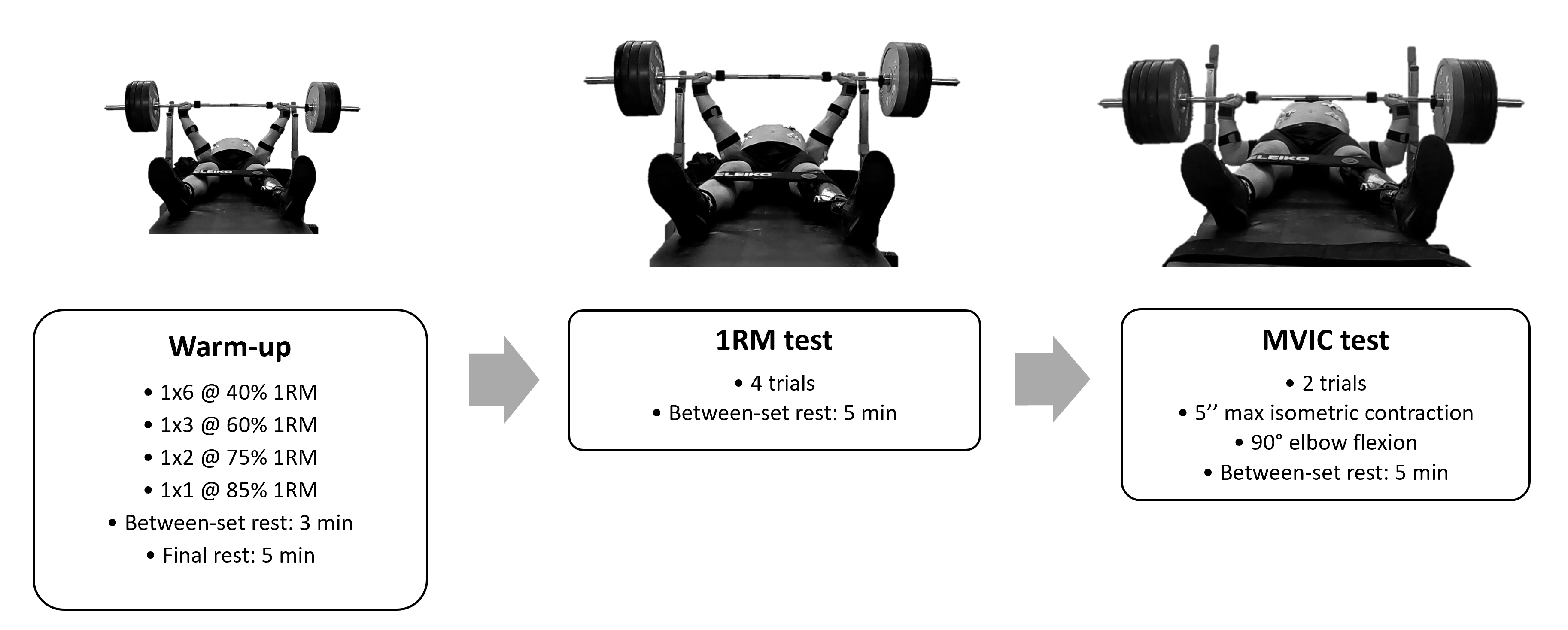

Supplement: Supplementary file 1 [file Image1.tif]
